# Editorial: Novel interventions for the prevention and control of communicable diseases

**DOI:** 10.3389/fpubh.2026.1796513

**Published:** 2026-02-18

**Authors:** Roland Salmon, George Karani, Daniel Rhys Thomas

**Affiliations:** 1 Consultant Epidemiologist, Cardiff, United Kingdom; 2 Environmental Health, Cardiff Metropolitan University, Cardiff, United Kingdom; 3 Communicable Disease Surveillance Centre, Public Health Wales, Cardiff, United Kingdom; 4 Applied Epidemiology, Cardiff Metropolitan University, Cardiff, United Kingdom

**Keywords:** behavioral science, communicable disease, epidemiology, new approaches, prevention

As early as 1998, World Health Day chose emerging infectious diseases, their challenges, and their solutions as its focus. This reflected the growing realization that the optimism of previous decades that infectious diseases were a problem of the past was hugely misguided. This optimism is frequently, and not altogether fairly, characterized by the oft-repeated 1960s quotation of the immunologist Frank Macfarlane Burnet, who went so far as to proclaim, together with his colleague David White, that “at least in the affluent West”, the grand objective had already been reached. “One of the immemorial hazards of human existence has gone,” he reported, because there is a “virtual absence of serious infectious disease today”. The World Health Organization also believed that the entire planet was ready to enter a new era by the end of the century. Meeting in Alma-Ata in 1979, the World Health Assembly adopted the “Health for All, 2000” (1) goal.

The prevention and control of communicable diseases had been brought about by dramatic societal improvements in housing, diet, and education. However, it was also achieved through a number of specific interventions that had become well established. Adequate sanitary and water engineering, control of insects and other disease vectors and widespread public acceptance of immunization all proved revolutionary in reducing the global burden of communicable disease.

Nevertheless, this optimism proved short-lived. The rapidly changing physical and social environment of the twenty-first century can, just as readily, drive the emergence or re-emergence of communicable diseases. The ongoing impacts of conflict and the movement of displaced people, the impact of climate change on vector distribution, changes in consumer behavior, including increasing mistrust in vaccines, and the relentless growth of antimicrobial resistance are all examples of these drivers. This requires fresh approaches to prevention and control. The development of a “one-health” approach has demonstrated the public health benefits of controlling zoonoses in animal populations, but at the same time, the potential for promoting antimicrobial resistance through the widespread veterinary use of treatments has been recognized. More locally, interventions such as contact tracing, post-exposure prophylaxis, isolation and hygiene advice, time-hallowed activities in acute health protection, may have to be modified dynamically in the light of emerging evidence of the changing epidemiology of communicable diseases. The recent emergence of mpox in Europe is an example of this. Similarly, during the early phase of the SARS-CoV-2 pandemic, non-pharmaceutical interventions (NPIs) such as physical distancing, handwashing, mask-wearing and contact tracing were scaled up to the level of the entire population and played various roles in the response in many countries, often without strong prior evidence of their effectiveness. What have we learnt about these interventions, and how has our learning during the pandemic translated into the prevention and control of other communicable diseases?

The United States' Centers for Disease Control and Prevention (CDC) provides guiding principles for interventions used in the field of epidemiological investigations of acute public health problems (2). These include guidance on the evaluation of surveillance systems and on the structuring of outbreak investigations. The CDC's Epidemic Intelligence Service (EIS) training programme, with its mix of short introductory courses and subsequent short teaching blocks, interspersed with practical experience at public health agencies, could be said to represent the model for Field Epidemiology Training Programmes (FETPs) worldwide, including the European Programme for Intervention Epidemiology Training (EPIET). This emphasis on when to implement interventions and the influence of uncertainty on decision-making are central to the “learning through doing” approach of modern FETPs, which is now adopted globally. However, while epidemiology plays a key role in the design and evaluation of interventions, so do other disciplines such as behavioral and environmental sciences. The selected articles in this Research Topic of Frontiers in Public Health describe the design and evaluation of new approaches to the prevention and control of communicable diseases in different regions of the world, settings and scientific perspectives, from front-line health protection to more theoretically based behavioral science and epidemiological approaches. The interventions described are largely those that are shown to prevent, reduce transmission or mitigate the effects of infection. The articles thus reflect aspects of a number of the United Nations' (UN) Sustainable Development Goals (3): goal 3 (good health and well-being), goal 9 (industry, innovation and infrastructure), goal 6 (clean water and sanitation), goal 11 (sustainable cities and communities), goal 12 (responsible consumption and production) and goal 17 (partnerships for the goals). Much of the innovation described draws, in turn, on the application of the two major fields of scientific progress that have characterized the end of the twentieth and the beginning of the twenty-first century: the digital information revolution [including artificial intelligence (AI)] and molecular biology (including genomics).

## The built environment

Some interventions apply existing knowledge intelligently. For example, Francisco and Watanabe constructed, in Mozambique, innovative and traditional houses and examined not only the efficacy of their prevention of mosquito entry, but also how liveable and thus acceptable the different designs were. Few parts of the built environment are as important for public health as wastewater systems, and in the United States, Xagoraraki et al. described an academia-industry-government collaboration that harnesses informatics and genomics in wastewater surveillance to generate epidemic intelligence in a timely fashion. In a more specific application of this approach, Jones et al. presented their findings from a mixed-methods evaluation of wastewater surveillance in prisons in Wales, UK, during the COVID-19 pandemic.

Similar to prisons, hospitals are important subsections of the built environment and can potentially be considered part of the therapeutic environment. Healthcare-acquired infections played an important role in the transmission of SARS CoV-2 and have historically been recognized as an important risk factor for influenza, pneumonia and the spread of antibiotic-resistant organisms. Li et al. (Huizhou, China) used a real-time, automatic nosocomial infection surveillance system to detect and address infections in the intensive care unit and throughout the studied hospital in a timely way. Similarly, Zhang et al. (Zhengzhou, China) used informatics to detect patients at high risk of contracting influenza in the hospital more effectively, permitting prevention and early intervention. Pacchiarini et al. (Wales, UK) looked at introducing whole genome sequencing in the context of COVID-19 and Clostridioides difficile disease management in healthcare settings. However, technical developments should not blind us to the role that can be played by trained human beings, particularly where access to technology may be more problematic. Woldeamanuel et al., in Ethiopia, in a project sponsored by the now sadly defunct United States Agency for International Development (USAID), assessed the effectiveness of a system of trained safety officers in preventing infections in healthcare facilities.

## The social environment

The widespread use of non-pharmaceutical interventions (NPIs) during the COVID-19 Pandemic has brought into focus the role that human behavior and the social environment play in the spread of disease and raised questions about the effectiveness of NPIs and their potential in the future. Li et al. used Susceptible-Exposed-Infectious-Recovered (SEIR) modeling to simulate the infections of different strains of COVID-19 under different scenarios in a hypothetical urban area of a prefecture-level city in Shandong Province, China, with a resident population of 2 million. In a similar vein, Guo et al. from the Centers for Disease Control and Prevention in the Northern Theater Command, Shenyang, China, used an individual-based computational model of infectious disease dynamics to simulate the regular, large-scale nucleic acid testing for community residents during an epidemic. The model assumed that when individuals tested positive, they and their household members would be isolated at home. Nevertheless, unlike empirical epidemiology, these modeling studies, which harness the huge data processing power of modern computers, are ultimately dependent on their models being accurate representations of the aspects of the world that are being studied.

Empirical epidemiology is also well represented. For example, in Nanjing, China, Liu et al. used genomic data from the field to comparatively analyse different discrimination methods to optimize the identification of Human Immunodeficiency Virus (HIV) clusters. Tebon et al. described how the collaborative “Sentinella” risk-based surveillance system was used in Northeast Italy in 2020 and 2021 to identify high-risk groups and to optimize early case detection. This enabled more efficient prevention and containment of potential clusters through strategic planning, particularly among the homeless. Also in Italy, in the Veneto Region, Milani et al. from the Regional Directorate of Prevention, Food Safety and Veterinary Public Health described their response to the World Health Organization's alert about the potential reintroduction of diphtheria and polio. This response involved geographical surveillance of immunization coverage, targeted community interventions to improve coverage, wastewater surveillance, and reinforcing the awareness of clinicians to enable the timely detection of potential cases. Alenezi et al., working for the Saudi Arabian Ministry of Health, described the use of virtual online post COVID-19 clinics to systematically examine the longer-term sequelae of SARS-CoV2 infection nationwide.

Similarly represented are empirical surveys of attitudes and behaviors, as these aspects of behavioral science have many similarities with epidemiological approaches. The role played by the knowledge and attitudes of pupils and parents in the prevention of influenza in Taicang, China, was described by Shi et al., as was the role of attitudes to COVID-19 vaccination on the uptake of influenza immunization, described by Zhang and Yi, in Shanghai. Toleha et al. (Dessie City, Ethiopia) documented a cross-sectional survey investigating the relationship between risk perception and knowledge of COVID-19 and protective practices.

Behavior in the online world and its impact on the prevention of real-world diseases are also covered. Giancotti et al., from Italy, conducted an umbrella review of systematic reviews published between 2011 and 2023, which showed the important role of social media in predicting and detecting disease outbreaks, potentially reducing morbidity and mortality through swift public health interventions. A research team from the Chinese People's Police University in Langfang examined, in some mathematical detail, the dynamic nature of the relationship between Baidu searches (the Chinese equivalent of Google) and cases of COVID-19. It is not fanciful to imagine these and similar findings being used to anticipate other public emergencies, including those beyond the field of infectious diseases, and to adapt the presentation of information to aid in the management of these emergencies.

## The therapeutic environment

Finally, in addition to the studies on healthcare-acquired infections (discussed above), there are two studies demonstrating how therapeutic innovations can have an impact at a community level. One study, from Germany, by Schmidt et al., documented newer approaches to HIV treatment with important implications for prevention. The other, from Spain, by Barbas Del Buey et al., described the impact of passive immunization with nirsevimab in reducing the community burden of Respiratory Syncytial Virus (RSV) in children.

## Conclusion

At a time when a new Pandemic Treaty has just been agreed at the World Health Assembly, this Research Topic of articles in this Research topic further demonstrates the current global interest in infectious diseases and their prevention. The contributions reflect some of modern science's favored technical approaches, such as genomics and mathematical modeling. However, when the various levels at which infectious diseases may be addressed are considered, there are some conspicuous omissions. Notably, there are no studies on climate change and deforestation, even though the role of these phenomena in changing the distribution of pathogens is well recognized. Similarly, no articles considered “One health” interventions targeting the public health impact of controlling disease in animal populations. Nevertheless, contributions were submitted from eight countries spanning four continents, with Australasia, South America and Antarctica absent. Eight contributions came from China, seven from Europe, three from Africa and just one from the United States. These reflect, therefore, science's current global social structure. Thus, China is now the source of the largest proportion of scientific articles published annually (4).

The potential of infectious diseases to change the trajectory of history has long been recognized (5, 6), and the COVID-19 Pandemic and the disruption that it caused have emphasized this and reignited the interest of historians. (7, 8). What this history emphasizes is that humanity needs to keep a sharp focus on infectious diseases, in the sorts of ways that these contributions describe. This is a task that is both important and difficult. It is also truly international.
